# Interaction of *Rickettsia felis* with histone H2B facilitates the infection of a tick cell line

**DOI:** 10.1099/mic.0.041400-0

**Published:** 2010-09

**Authors:** Chutima Thepparit, Apichai Bourchookarn, Natthida Petchampai, Steven A. Barker, Kevin R. Macaluso

**Affiliations:** 1 Department of Pathobiological Sciences, Louisiana State University, School of Veterinary Medicine, Baton Rouge, LA 70803, USA; 2 Department of Comparative Biomedical Sciences, Louisiana State University, School of Veterinary Medicine, Baton Rouge, LA 70803, USA; 3 Department of Technology and Industries, Faculty of Science and Technology, Prince of Songkla University, Pattani 94000, Thailand

## Abstract

Haematophagous arthropods are the primary vectors in the transmission of *Rickettsia*, yet the molecular mechanisms mediating the rickettsial infection of arthropods remain elusive. This study utilized a biotinylated protein pull-down assay together with LC-MS/MS to identify interaction between *Ixodes scapularis* histone H2B and *Rickettsia felis*. Co-immunoprecipitation of histone with rickettsial cell lysate demonstrated the association of H2B with *R. felis* proteins, including outer-membrane protein B (OmpB), a major rickettsial adhesin molecule. The rickettsial infection of tick ISE6 cells was reduced by approximately 25 % via RNA-mediated H2B-depletion or enzymic treatment of histones. The interaction of H2B with the rickettsial adhesin OmpB suggests a role for H2B in mediating *R. felis* internalization into ISE6 cells.

## INTRODUCTION

Ticks serve as biological vectors for a variety of disease-causing agents, including protozoa, viruses and bacteria. Unique among the tick-borne bacteria is the close association between ticks and members of the genus *Rickettsia*. Ixodid ticks can serve as a horizontal transmission vector for particular species such as *Rickettsia rickettsii*, the aetiological agent of Rocky Mountain spotted fever, or as a vertical transmission reservoir for rickettsial endosymbionts such as *Rickettsia peacockii*. While rickettsiae vary in their pathogenicity to vertebrate and tick hosts, their requirement for the arthropod vector is unequivocal; however, the infection process within the arthropod remains poorly defined.

In contrast to the dearth of knowledge of tick–*Rickettsia* interactions, the molecular interaction between rickettsiae and vertebrate cells has been steadily investigated. Studies examining rickettsial attachment to, and invasion of, vertebrate cells *in vitro* have identified receptor-mediated endocytosis as the mechanism for rickettsial infection. Prior to fusion of the endosome and lysosome, rickettsiae utilize phospholipase D and haemolysin to escape from the endosome (Whitworth *et al.*, 2005). Once in the cytosol, rickettsiae replicate (Weiss, 1982) and some species invade the nucleus (Silverman & Wisseman, 1979). Successive infections occur by host cell lysis and the infection of new cells, or via recruitment of host-derived actin and the assemblage of actin tails that facilitate the intracellular movement of rickettsiae through the cytoplasm, across the cell membrane and into neighbouring cells (Hackstadt, 1996; Heinzen *et al.*, 1993, 1999). More recent studies have utilized a membrane protein–protein pull-down assay to illuminate the molecular mechanisms of rickettsial infection in vertebrate cells, identifying the vertebrate molecule Ku70, a subunit of a DNA-dependent protein kinase, as a host cell receptor for *Rickettsia conorii*, and demonstrating the cascade process of *R. conorii* internalization into nonphagocytic mammalian cells, comprising receptor binding, signal transduction and cytoskeletal rearrangement (Martinez *et al.*, 2005). Specifically, the adherence of *R. conorii* outer-membrane protein B (OmpB) to Ku70 on Vero cells stimulates the ubiquitination of Ku70 by ubiquitin ligase. Subsequent signal transduction activates the Arp2/3 complex, coordinating actin rearrangement and the resultant bacterial endocytosis (Martinez *et al.*, 2005; Martinez & Cossart, 2004).

A number of tick-derived molecules have been identified as being responsive to rickettsial infection (Macaluso *et al.*, 2006). Using a number of techniques, including differential display PCR (Macaluso *et al.*, 2003), subtractive suppression hybridization (Mulenga *et al.*, 2003) and homologue cloning (Simser *et al.*, 2004), the constituents of the tick–*Rickettsia* interchange are being identified. With the advent of tools including tick-derived cell culture and low-passage rickettsial isolates, further opportunities to examine many of these interactions *in vitro* now exist (Munderloh & Kurtti, 1995). In the current study, a pull-down assay was utilized to identify molecules that associate with rickettsiae. Subsequently, RNA-mediated interference (RNAi) and infection bioassays were employed to characterize interactions between ticks and rickettsial molecules. Elucidating the molecular relationship between arthropods and rickettsiae is essential in deciphering the basis of vector competence, transmission events, and the epidemiology of emerging and re-emerging rickettsial diseases.

## METHODS

### Tick cell and *Rickettsia* cultivation.

*Ixodes scapularis*-derived ISE6 cells were cultivated in L-15B medium (Gibco-BRL) supplemented with 10 % fetal bovine serum (FBS) (HyClone) and 10 % tryptose phosphate broth (Sigma), pH 6.8–7.0, with 5 % CO_2_ at 32 °C (Munderloh & Kurtti, 1989). *Rickettsia felis* (LSU strain) was propagated in ISE6 cells as previously described (Pornwiroon *et al.*, 2006) and harvested when the infected cells were around 80–90 % confluent. For rickettsial infection inhibition assays, low-passage *R. felis* cultures were partially purified as described by Sunyakumthorn *et al.* (2008). For pull-down and co-immunoprecipitation assays, *Rickettsia* cells were purified by Renografin density-gradient centrifugation to remove host-derived protein contamination as described previously (Pornwiroon *et al.*, 2009). Rickettsial viability and enumeration were assessed as previously described (Sunyakumthorn *et al.*, 2008).

### Pull-down assay.

ISE6 cells were surface-labelled with Sulfo-NHS-LC-Biotin (Pierce) for 30 min on ice. The biotinylated cells were sonicated in ice-cold 0.1 % NP-40/PBS buffer. The insoluble materials were discarded after centrifugation and the protein extracts in the soluble fraction were incubated with Renografin-purified *R. felis* for 12 h at 4 °C. *Rickettsia*-bound protein pellets were collected, washed, and eluted using 0.1 % NP-40/PBS with 1 M NaCl for 1–1.5 h at 4 °C. Eluates were analysed on Bistris gels (Invitrogen) with Western blotting analysis using peroxidase-conjugated streptavidin. For a negative control, *Escherichia coli* (Invitrogen TOP10 *E. coli* strain, similar to the DH10B strain) was used in the assays instead of purified *R. felis.* Grown in LB broth for 16 h, *E. coli* was collected by centrifugation at 3300 ***g*** for 10 min, washed twice with PBS and then used in the pull-down assays.

### Protein identification.

The protein bands of interest were identified by LC-MS/MS as previously described (Sunyakumthorn *et al.*, 2008). Searches were performed in the NCBInr and Swiss-Prot databases using a Mascot search via http://www.matrixscience.com.

### Gel electrophoresis and Western blotting.

Cell lysates or protein extracts were subjected to electrophoresis through either 4–12 % or 12 % Bistris gels and transferred to a PVDF membrane. The membrane was blocked with 5 % skim milk in TBS-T at room temperature for 1 h. Primary antibodies were diluted 1 : 5000 for *α*-histone H2B (ab1790; Abcam) and 1 : 400 for *α*-tubulin (B-5-1-2) (sc-23948, Santa Cruz Biotechnology). HRP-conjugated secondary antibodies, goat anti-rabbit IgG (A-6154, Sigma), and goat anti-mouse IgG (Pierce) were used at 1 : 10 000 dilution. The signal was developed by using a chemiluminescent substrate kit (Pierce).

### Quantitative real-time PCR (qPCR).

The copy numbers of rickettsial and tick genes were established by qPCR as recently published (Zanettii *et al.*, 2008). Infectivity was quantified in terms of rickettsiae per host cell by a qPCR using specific primers for the single copy of the rickettsial 17 kDa antigen gene and the tick calreticulin gene. A plasmid containing portions of both genes was constructed as described by Zanettii *et al.* (2008), and used as a standard for template quantification. The percentage of infection was determined designating the infectivity (the quantity of rickettsiae per host cells) of the control group as 100 %.

### RNA-mediated interference of tick gene transcription.

dsRNA specific to the full-length gene for tick histone H2B was synthesized from ISE6 RNA by RT-PCR. Synthesized from ISE6 total RNA, the full-length gene for histone H2B was amplified from the first-strand cDNA by using a pair of specific primers containing the T7 promoter sequence at the 5′ end. Primer sequences are 5′-TTA ATA CGA CTC ACT ATA GGG CGC ACG TCC GAC AAG AAG AAG AAA-3′ and 5′-TTA ATA CGA CTC ACT ATA GGG TTG AAG AAG TGT ACT TCG TGA CCG-3′. The PCR product was transcribed into dsRNA based on the T7 RNA polymerase promoter sequence by using a MEGAscript T7 kit (Ambion). Scramble dsRNA was synthesized, using a pTRI-Xef DNA template provided in the kit, for use as a negative control. dsRNA specific to the gene for histone H2B or scramble dsRNAs were introduced into ISE6 cells by using RNAiFect transfection agent (Qiagen). In parallel, ISE6 cells were transfected with fluorescently labelled dsRNA (Cy3 siRNA Labelling kit, Ambion) to assess the transfection efficiency and subcellular localization of dsRNA. To determine the post-transfection period at which the targeted protein expression was depleted, the transfected cells were collected daily to assess the regulation of gene transcription, by RT-PCR, and protein expression, by Western blotting.

### Infection inhibition assays.

ISE6 cells grown on 48-well plates were transfected with or without 14 nM dsRNAs targeting histone H2B or scramble genes. The infection inhibition assay was performed on the day post-transfection on which the reduction of the targeted protein was observed as described earlier. The treated cells were infected with partially purified *R. felis* at an m.o.i. of 200 at 32 °C for 90 min. Then unbound *R. felis* was removed and the infected cells were washed twice with PBS prior to collection. Genomic DNA was extracted from the samples to assess the rickettsial and tick gene number by qPCR. The assays were performed in triplicate. For carboxypeptidase B (CpB) treatment, ISE6 cells were pre-treated with 0, 10, or 50 units of CpB (Worthington) in serum-free L-15B medium at 37 °C for 60 min prior to infection with *R. felis*.

### Co-immunoprecipitation (Co-IP).

Histones were extracted from ISE6 cells by lysis with Triton Extraction Buffer (TEB: PBS containing 0.5 % Triton X-100 and protease inhibitor) on ice for 10 min with gentle stirring. Cell pellets were collected, washed with TEB buffer and then incubated with 0.2 M HCl for 16 h at 4 °C. Histones in the supernatant fraction were harvested by centrifugation at 380 ***g*** for 10 min at 4 °C. The Renografin-purified *R. felis* cells were lysed in 1 % NP-40 buffer; the protein was quantified by Bradford assay and 100 μg was pre-incubated with 100 μg of the acid-extracted histone at 4 °C overnight. The *Rickettsia*–H2B complex was captured and insolubilized by incubation with 2 μg of polyclonal antibody against histone H2B followed by the antibody-binding protein G (Santa Cruz Biotechnology). The Co-IP products were collected by centrifugation at 600 ***g*** for 5 min and washed in 1 % NP-40 prior to gel electrophoresis and Western blotting.

### Histone-mediated rickettsial entry assay.

The partially purified *R. felis* cells were pre-incubated with or without the acid-extracted histone from ISE6 cells (2–10 μg) in a total volume of 500 μl for 1 h at 32 °C prior to infection (m.o.i. of 200) of the ISE6 cells pre-grown on a 96-well tissue culture plate. After infection for 90 min at 32 °C, rickettsial inocula were removed and the infected cells were collected for DNA extraction. Numbers of rickettsiae per host cell were determined by qPCR as mentioned earlier. To assess the role of the C-terminal portion of the histone in rickettsial entry mediation, the extracted histone was pre-treated with 50 units of CpB at 37 °C for 60 min. The enzymic reaction was stopped by addition of HCl to a final concentration of 0.05 M. The partially purified *R. felis* cells were incubated with the CpB-treated or untreated histone prior to infecting (m.o.i. of 100) ISE6 cells pre-grown on coverslips. After infection for 90 min at 32 °C, rickettsial inocula were removed and the *Rickettsia*-associated cells were analysed by immunofluorescence assay.

### Immunofluorescence assay.

Uninfected and *R. felis*-infected ISE6 cells, pre-grown on coverslips, were fixed with 4 % paraformaldehyde in PBS and permeabilized with 0.5 % Triton X-100 in PBS. To detect *R. felis*, the fixed cells were stained with mouse anti-*R. felis* serum (1 : 200), followed by staining with FITC-conjugated anti-mouse IgG antibody (1 : 200, KPL). Slides were examined by fluorescence microscopy using the ×63 oil objective.

### Statistical analysis.

The raw data and ranked data were analysed by one-way ANOVA with repeated measurement, followed by post hoc tests, for which both Tukey's HSD test and the Ryan–Einot–Gabriel–Welsch test were performed using the SAS GLM procedure. For all comparisons, a *P*-value≤0.05 was taken as significant.

## RESULTS

### Binding of *R. felis* to ISE6 cell-surface histone H2B

**Fig. 1.**
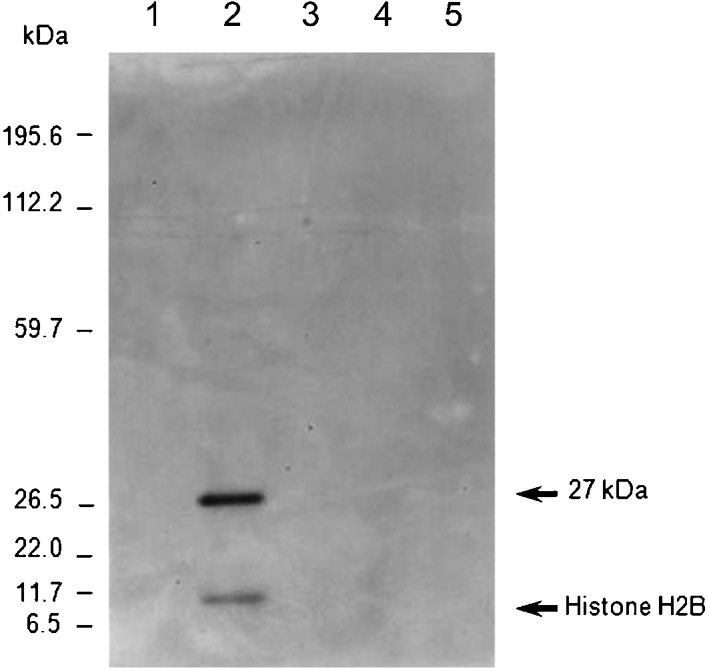
*R. felis* interacts with ISE6 cell surface proteins. Western blotting analysis of eluates from pull-down assays using biotinylated ISE6 surface proteins and/or Renografin-purified *R. felis*. Biotinylated ISE6 cells in 0.1 % NP-40/PBS buffer were incubated with or without purified *R. felis. Rickettsia*-bound protein pellets were collected, washed, and eluted using 0.1 % NP-40/PBS with 1 M NaCl. Eluates were analysed on Bistris gels with Western blotting analysis using peroxidase-conjugated streptavidin. Lanes 1, labelled ISE6 protein; 2, labelled ISE6 protein+*R. felis*; 3, unlabelled ISE6 protein; 4, unlabelled ISE6 protein+*R. felis*; 5, *R. felis*.

**Fig. 2.**
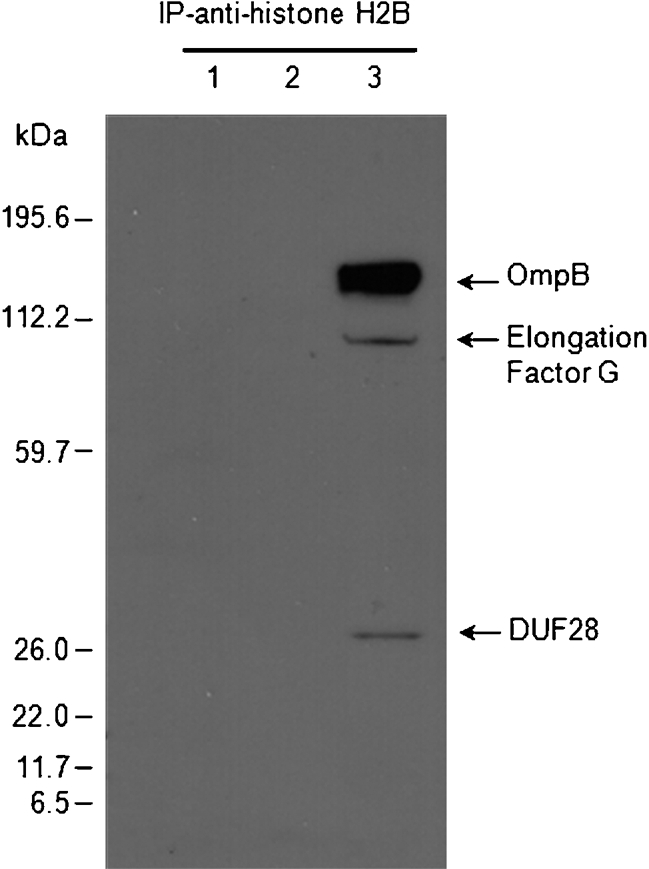
*R. felis* OmpB protein binds ISE6 histone H2B. Histone extract from ISE6 cells was pre-incubated with or without Renografin-purified *R. felis.* The *Rickettsia*–H2B complex was captured and insolubilized by sequential incubation with polyclonal antibody against histone H2B and antibody-binding protein G. The Co-IP products were collected and analysed on Bistris gels by Western blotting using mouse anti-*R. felis* serum. Lanes 1, ISE6 histone extract; 2, purified *R. felis* cell lysate; 3, lysates of ISE6 histone extract and the purified *R. felis*. Corresponding proteins were identified by LC-MS/MS.

A bacterial affinity pull-down assay was employed to assess rickettsial interaction with the ISE6 cell-surface proteins. The cell-surface proteins of intact ISE6 cells were selectively labelled with biotin prior to incubation with purified *R. felis*. The rickettsia-bound proteins were eluted, and analysed on Bistris gels with Western blotting analysis using peroxidase-conjugated streptavidin. Two putative *Rickettsia*-binding proteins, with approximate sizes of 14 and 27 kDa, specifically bound to purified *R. felis* (Fig. 1). The specificity of the *Rickettsia*–arthropod interaction was confirmed when the 14 and 27 kDa proteins were not detected when using *E. coli* instead of *Rickettsia* as a control for the pull-down assay (Supplementary Fig. S1, available with the online version of this paper). The trypsin-digested peptides of the 14 kDa band analysed by LC-MS/MS significantly matched *I. scapularis* histone H2B (GenBank accession no. AAY66901). Peptide analysis of the 27 kDa band identified a parvulin-like peptidyl-prolyl *cis-trans* isomerase (PPIase) (GenBank accession no. YP_246958), one of the outer-membrane proteins of *R. felis*. The interaction of *R. felis* with tick histone H2B was confirmed by co-immunoprecipitation. Histone extracted from ISE6 cells was incubated with *R. felis* protein lysate prior to incubation with antibody against histone H2B and coupling with antibody-binding protein-G. As shown in Fig. 2, Western blotting analysis of the Co-IP eluates probed with mouse anti-*R. felis* revealed that three bands of *R. felis* proteins coeluted with a histone–*Rickettsia* complex. Mass-spectometry analysis of trypsin-digested peptides identified two minor bands, which matched to *R. felis* elongation factor G (GenBank accession no. Q8KTA8) and the hypothetical protein DUF28 with unknown function (GenBank accession no. YP_246815), and a major band of approximately 120 kDa, identified as *R. felis* OmpB (GenBank accession no. AAY61056). The histone H2B band was detected in the Western blotting membrane when reprobed with antibody against H2B to confirm the presence of H2B in the histone–*Rickettsia* binding complex (data not shown).

### RNA-mediated interference (RNAi) of tick gene transcription

**Fig. 3.**
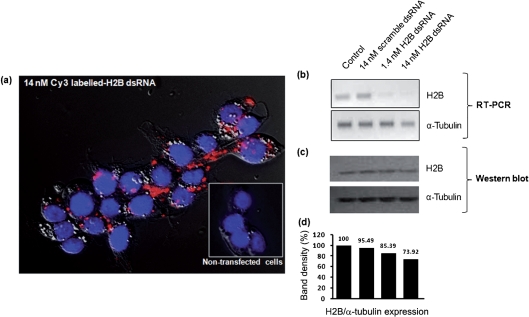
Transcriptional and translational silencing of tick histone H2B using dsRNA in ISE6 cells. ISE6 cells were transfected with dsRNA as indicated. (a) The transfection efficiency and cellular localization of dsRNA was observed at 24 h post-transfection by detection of Cy3-labelled dsRNA in the transfected cells under fluorescence microscopy. Cell nuclei were stained with DAPI. Red fluorescence represents localization of dsRNA. At day 11 post-transfection, regulation of histone H2B transcripts and protein was observed. (b) RT-PCR detection of histone H2B and *α*-tubulin transcripts of the dsRNA-transfected cells. (c) Western blotting analysis using anti-histone H2B antibody. The membrane was reprobed with anti-*α*-tubulin antibody. (d) Densitometry of histone H2B and *α*-tubulin Western blotting. Band density was analysed using Quantity One analysis software (Bio-Rad). The density of histone H2B was normalized with *α*-tubulin. The bar graph shows the expression of histone H2B relative to that of the control (100 %).

To further delineate the role of histone H2B in the rickettsial infection of tick cells, we utilized RNAi to deplete cells of H2B. In the current study, RNAi methodology was adapted to ISE6 cells to knock down transcription and translation of the putative *R. felis*-binding molecule histone H2B. ISE6 cells were either mock-transfected with only transfection buffer (negative control), transfected with dsRNA targeting the full-length gene for the tick histone H2B, or transfected with scrambled nucleotides (non-specific control). The transfection efficiency and subcellular localization of dsRNA were verified at 24 h post-transfection by detection of fluorescently labelled dsRNA under fluorescence microscopy (Fig. 3a). The transfected cells were collected daily to assess the gene transcription and protein regulation of the gene/protein of interest. The introduction of 1.4 and 14 nM dsRNA, targeting H2B production specifically, depleted the targeted gene transcription as early as 3 days post-transfection (data not shown), with silencing persisting through to day 11 (Fig. 3b). The knocked-down transcript was recovered at day 13 post-transfection (data not shown). Although the silencing effect at the translational level could not be clearly observed in the early period post-transfection, at day 11 post-transfection a reduction of H2B protein in the cells transfected with 14 nM dsRNAs was observed (Fig. 3c). The altered regulation of H2B expression was normalized with *α*-tubulin and the band intensity was quantified by densitometry analysis (Fig. 3d). Relative to the mock-transfected control, H2B expression was reduced by approximately 26 % and 15 % in the cells transfected with dsRNA targeting H2B at 14 nM and 1.4 nM, respectively. The induction of the scramble dsRNA did not affect the regulation of H2B at either the transcriptional or the translational level (Fig. 3b, c, d).

### Histone H2B is involved in the infection of tick cells by *R. felis*

**Fig. 4.**
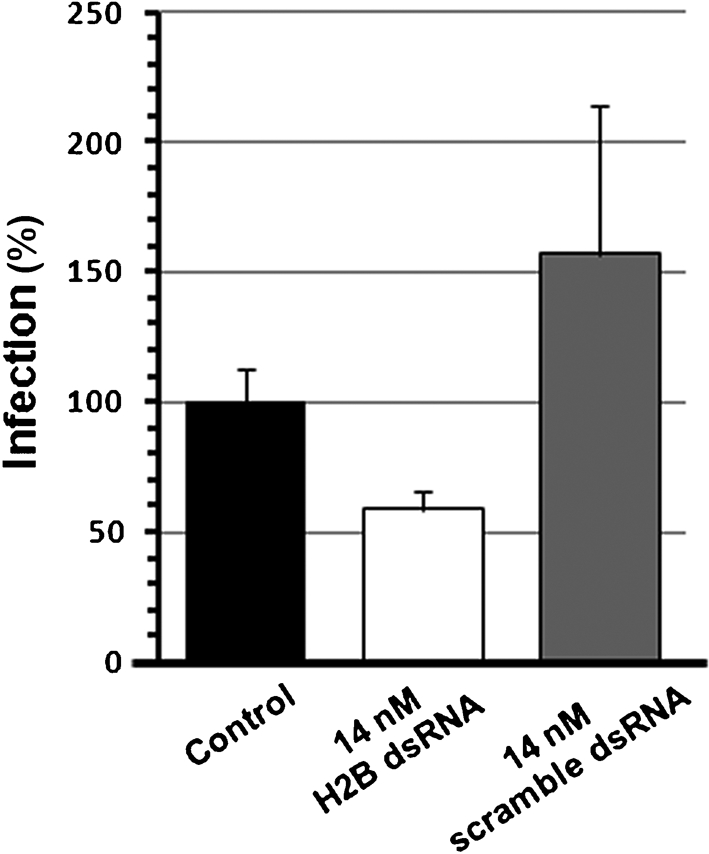
RNAi-mediated depletion of host cell histone decreases rickettsial infection. The infection bioassay was performed when histone H2B was depleted in ISE6 cells (11 days post-transfection). ISE6 cells (control), histone H2B-depleted cells (14 nM H2B dsRNA), or mock-transfected cells (14 nM scramble dsRNA) were infected (m.o.i. 200) with partially purified *R. felis* for 90 min at 32 °C. Unbound *R. felis* was removed and infected cells were washed with PBS prior to collection. Infectivity was calculated (rickettsial gene/host gene) by qPCR and is shown as a percentage of the control value. Bars represent the mean (±sem) of three individual assays.

**Fig. 5.**
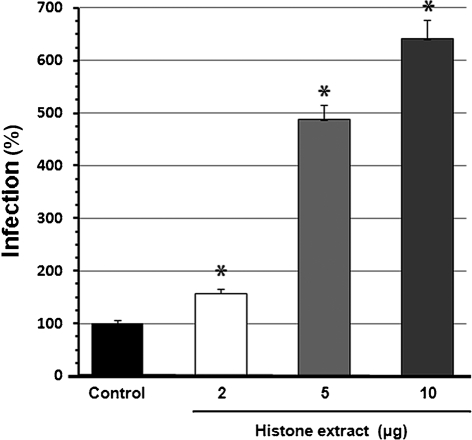
Histone enhances *R. felis* entry into tick cells. Partially purified *R. felis* was pre-incubated with tick-derived histone (2–10 μg) for 1 h at 32 °C prior to infection (m.o.i. 200) of ISE6 cells. After 90 min, rickettsial inocula were removed and cells were collected. Infectivity was calculated (rickettsial gene/host gene) by qPCR. The bar graph represents percentage of infection relative to the control (as 100 %). Bars represent the mean (±sem) of three individual assays. Asterisks represent a significant difference from the control.

To determine if the putative *R. felis*-binding protein histone H2B is functionally involved in the infection of tick cells, the infectivity of *R. felis* in the dsRNA-mediated-H2B-depleted ISE6 cells was assessed. The reduction of H2B expression by dsRNA-mediated silencing inhibited *R. felis* infection by approximately 61 % relative to the control. The infectivity of *R. felis* was variable in the scramble dsRNA-treated cells; however, there was no inhibitory effect on rickettsial infectivity (Fig. 4). In a separate bioassay, the histone-mediated infection of tick cells by rickettsiae was assessed. Pre-incubation of partially purified *R. felis* with the histones extracted from ISE6 cells could facilitate rickettsial infection in a dose-dependent manner, as shown by the significantly increased number of infected cells in the histone pre-incubation groups (Fig. 5).

### A carboxy-terminal region of histone H2B is involved in the infection of tick cells by *R. felis*

**Fig. 6.**
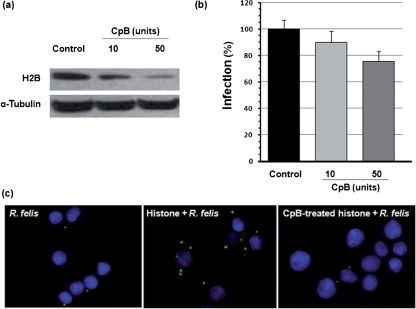
Carboxypeptidase treatment decreases rickettsial infection. ISE6 cells were pre-treated with various concentrations of CpB at 37 °C for 1 h prior to infection (m.o.i. 200) with partially purified *R. felis* for 1 h at 32 °C. (a) The effectiveness of CpB in cleavage of C-terminal histone H2B on ISE6 cells was determined by Western blotting analysis using anti-histone H2B antibody with anti-*α*-tubulin antibody as a load control. (b) Infectivity of *Rickettsia* on the CpB treated/untreated ISE6 cells as calculated (rickettsial gene/host gene) by qPCR. The bar graph represents percentage infection relative to the control (as 100 %). Bars represent ±sem of three individual assays. (c) ISE6 cells were infected (m.o.i. 100) with *R. felis* alone, *R. felis* pre-incubated with 10 μg histone, or *R. felis* pre-incubated with 50 units CpB-treated histone. After 90 min, inocula were removed and cells were fixed and permeabilized. Rickettsiae were detected by immunofluorescence assay and cell nuclei were stained with DAPI. Lower numbers of rickettsiae were observed in the CpB-treated preparations.

Histone H2B is rich in basic amino acids lysine and arginine at its C terminus. C-terminal lysyl residues of H2B were previously reported to serve as a plasminogen-binding site (Das *et al.*, 2007; Herren *et al.*, 2006; Miles *et al.*, 1991). To determine if the C-terminal basic amino acid region of H2B is involved in *R. felis* infection, ISE6 cells were treated with CpB to remove the basic amino acids, including lysine, arginine and ornithine, from the C terminus of polypeptides prior to infection with *R. felis*. The effectiveness of CpB in cleavage of the C-terminal region of histone H2B on ISE6 cells was determined by Western blotting with antibody raised against the C terminus of histone H2B. Of the varying CpB concentrations used, a saturation effect was observed on the ISE6 cells treated with 50 units of CpB as shown by the least amount of H2B remaining intact (Fig. 6a). Increasing CpB concentration to 100 units did not result in additional cleavage of H2B; therefore, the infection inhibition assay was done with the ISE6 cells treated with or without 10 or 50 units of CpB. The infectivity of *R. felis* in the CpB-treated ISE6 cells was compared to the control cells. Fig. 6(b) shows a dose-dependent inhibitory effect of CpB treatment on rickettisal infection. The C-terminal portion of the histone contributes to rickettsial infection, as CpB treatment of the ISE6 cells inhibited *R. felis* infection by approximately 10 % and 25 % relative to the control for 10 and 50 units, respectively. To determine if the inhibitory effect of CpB treatment is specific to the C-terminal lysine residues of histone, resulting in rickettsial infection inhibition, histone was treated with 50 units CpB to cleave the C-terminal basic amino acids before incubation with the partially purified *R. felis*. Either *R. felis* alone or *R. felis* with CpB-treated/untreated histone were incubated with ISE6 cells and infected cells were analysed by immunofluorescence assay (Fig. 6c). Corresponding to the ability of histone to mediate rickettsial entry (Fig. 5), a marked accumulation of rickettsiae in the histone pre-incubation group was observed compared to *R. felis* alone. Rickettsial accumulation declined in the CpB-treated histone pre-incubation group suggesting that the C-terminal basic amino acid residues serve as a *Rickettsia*-binding region or participate in mediation of rickettsial entry.

## DISCUSSION

The initial interaction between *Rickettsia* and the arthropod host is a critical determinant of successful rickettsial infection and transmission; however, progress made in identifying host molecules facilitating or involved in rickettsial infection is lacking. This study thus sought to identify and characterize arthropod-derived molecules involved in the rickettsial infection of arthropod host cells. The utilization of the pull-down assay was intended to identify arthropod-derived proteins that bind rickettsiae. The specificity of the reaction was verified by a control reaction using *E. coli*, which did not result in the identification of the same bands. Although *R. felis* is typically associated with insect hosts, there is molecular evidence of *R. felis* in acarine hosts such as ixodid ticks (reviewed by Reif & Macaluso, 2009). The isolation of *R. felis* (strain LSU) in the *I. scapularis* (ISE6) cell line allows assessment of a low-passage rickettsial isolate; however, the association of identified molecules should be confirmed in *Rickettsia*–arthropod relationships observed in nature.

Interestingly, the rickettsial parvulin-like PPIase protein produced a detectable streptavidin/biotin signal in the pull-down assay. The occurrence of rickettsial parvulin-like PPIase in the tick–*Rickettsia* pull-down eluates suggests an association of this protein with the *Rickettsia*–host cell surface protein interaction. Although the identification of a parvulin-like PPIase was unanticipated, it generates interest regarding potential functional activity in rickettsial infection. Bacterial PPIase-containing proteins have been found to be involved in protein trafficking, arrangement or maturation of outer-membrane proteins, and bacterial virulence (Behrens *et al.*, 2001; Emelyanov & Demyanova, 1999). With respect to bacterial infection, it was proposed that parvulin-like PPIases may alter the conformation of either bacterial or host cell surface components, facilitating adherence between bacteria and host cells (Emelyanov & Demyanova, 1999). The involvement of PPIase-containing proteins in host cell adherence was clearly demonstrated in other bacterial infections. For example, inactivation of the PPIase domain of the Mip protein of *Legionella pneumophila*, and the Mip-like protein of *Chlamydia trachomatis*, both of which share similar PPIase activity, interfered with the initiation of bacterial infection of mammalian cells (Lundemose *et al.*, 1993). Although not of tick origin, the detection of this rickettsial outer-membrane protein, possessing putative adhesion activity in the host cell binding complex, suggests it has a role in the *Rickettsia*–host interaction, and that it is of interest for further investigation.

A tick-derived molecule, histone H2B, was also identified via the *Rickettsia* and tick cell surface protein interaction pull-down assay. Histone H2B, one of the core histones forming the nucleosome complex with the linker histone H1, primarily locates and functions in the cell nucleus (Baake *et al.*, 2001; Khan *et al.*, 1998). However, consistent with the current study, there is increasing evidence that histone proteins localize in other cellular compartments, for example in the cytoplasm and on the cell surface of both eukaryotic and prokaryotic cells (Bolton & Perry, 1997; Das *et al.*, 2007; Herren *et al.*, 2006; Holers & Kotzin, 1985; Khan *et al.*, 1998; Qiu & Wang, 2008; Zlatanova *et al.*, 1990). Histone H2B lacks a transmembrane domain (Herren *et al.*, 2006); a clear translocation process from nucleus to cell membrane has not been determined. However, histone H2B in the form of DNA-core histone complexes has been identified on activated T-cell membranes anchoring to a heparan sulfate proteoglycan (Watson *et al.*, 1999). The association between a tick-derived nuclear protein such as histone H2B and rickettsiae is not surprising, as evidence of the receptor function of typical non-cell surface proteins is increasing. For example, Ku70 and its heterodimer, Ku80, are typically retained in the nucleus and play several roles, including the repair of double-strand breaks by joining the DNA ends, facilitating the coordination of other DNA repair proteins (Featherstone & Jackson, 1999). In an undefined process, Ku70 translocates from the nucleus to the cytoplasm and also to the plasma membrane, where it is multi-functional (Amsel *et al.*, 2008; Prabhakar *et al.*, 1990; Tai *et al.*, 2002).

Rickettsial invasion initiates with the bacteria binding a molecule(s) on the cell surface of the host cell. The Co-IP results demonstrated the interaction of rickettsial proteins with tick histone H2B. Among these proteins, three rickettsial antigenic proteins were recognized by mouse anti-*R. felis*; these include elongation factor G, the hypothetical protein DUF28, and OmpB. OmpB, the most abundant immunogenic surface protein of *Rickettsia*, is one of the crystalline bacterial cell surface layer (S-layer) proteins, with known functions in cell adhesion and surface recognition (Zlatanova *et al.*, 1990) in vertebrates, and specific interaction with the mammalian DNA-binding molecule Ku70. Although outer-membrane proteins are antigenically specific among various rickettsial species (Anacker *et al.*, 1987), the critical role in host cell invasion seems conserved (Li & Walker, 1998; Martinez *et al.*, 2005; Uchiyama *et al.*, 2006). Other rickettsial molecules have been identified as adhesins or are associated with infection, including outer-membrane protein A (OmpA). The gene encoding OmpA is present in *R. felis* and is commonly found in the members of the spotted fever group of *Rickettsia*; however, *R. felis ompA* possesses premature stop codons (Zavala-Castro *et al.*, 2005). The truncated *ompA* gene encodes the putative OmpA protein, which lacks the *β*-barrel autotransporter fragment, resulting in the inability to export molecules to the outer membrane (Zavala-Castro *et al.*, 2005). Rickettsial infection despite a nonfunctional OmpA suggests that redundant mechanisms of rickettsial adhesion to the host cell exist.

Effectively applied RNAi can facilitate the study of proteins *in vivo* and *in vitro* for several species of tick (Kurtti *et al.*, 2008; Ramakrishnan *et al.*, 2005). In the current study, an inhibitory effect on *R. felis* infection was observed in the absence of a complete knock-down of histone H2B protein. RNA-mediated interference has been effectively applied to study the functions of several proteins in various cells, albeit with limited effectiveness for some proteins. While dsRNA targeting H2B could efficiently and immediately knock down histone mRNA, the H2B protein was not completely depleted. The impaired effect of dsRNA-mediated silencing at the protein level might occur because of a low turnover rate for the histone protein. Although histone mRNA reportedly has a half-life as short as 10 min to several hours (Borun *et al.*, 1967; Marzluff & Duronio, 2002), the rate of histone protein turnover was estimated to be relatively slow [estimated half-life 30 h: computational analysis by ProtParam (Gasteiger *et al.*, 2005)]. In addition, histone protein is abundantly synthesized and accounts for 30 % of total protein synthesis (Kedes, 1979). Consistent with the results of the current study, the delayed effect of RNAi on protein expression in tick cell culture (ISE6) was also noted by Kurtti *et al.* (2008), in which decreases in detectable protein expression began on day 7 with the lowest levels of expression detected at day 21 post-transfection. Likewise, in another *I. scapularis* cell line, IDE8, while some of targeting genes were partially silenced by RNAi, the silencing effect on protein level was not provided (de la Fuente *et al.*, 2007). Compared to *in vivo* RNAi in ticks (Ramakrishnan *et al.*, 2005), it is not clear if the mechanisms of RNAi are slow due to the target molecule, or due to the origin of the host cell (cell culture versus whole tick).

The specificity of histone H2B residues participating in rickettsial binding was further verified via enzyme-mediated histone modification. Histone H2B possesses a basic amino-acid-rich region at its C terminus, composed of lysine and arginine residues. C-terminal lysyl residues of H2B were demonstrated to serve as a plasminogen recognition binding site (Das *et al.*, 2007; Herren *et al.*, 2006; Miles *et al.*, 1991) and the blocking of H2B with an antibody specific to its C-terminal octopeptide, including the cleavage of its C-terminal lysyl residues by CpB, abolished the binding of plasminogen to the cells (Herren *et al.*, 2006). The loss of infectivity of rickettsiae on the CpB-treated ISE6 cells implies the involvement of these C-terminal, positively charged residues of H2B in *Rickettsia* binding. Interestingly, the lysine-rich C-terminal region was also reported as the binding region on histone H1, which serves as the specific receptor for the adhesin of enterotoxigenic *E. coli* (Zhu *et al.*, 2005). The involvement of H2B in the rickettsial infection of arthropod host cells was confirmed *in vitro* by different approaches. The study demonstrates that in ISE6 cells the partial depletion of H2B by RNAi inhibits rickettsial infection and the cleavage of the C-terminal region of H2B by CpB limits histone-mediated infection. Although no single method completely abrogated the activity of H2B in tick cells, the partial inhibition of infection observed in both bioassays demonstrates a role for H2B in the infection of arthropod cells by *R. felis*, suggesting redundant mechanisms of infection are involved. Additionally, the ability of histone to mediate rickettsial entry into the cells confirms the *Rickettsia*–histone interaction and supports the involvement of histone in rickettsial infection. While we have demonstrated that H2B interacts directly with *R. felis* and facilitates rickettsial infection, we cannot exclude the potential role of histones in serving as an intermediary between rickettsiae and an additional host factor. The fact that the addition of increasing concentrations of isolated histone increased rickettsial infectivity, as opposed to competitively inhibiting *Rickettsia*–host cell association suggests that histones may act either as a direct rickettsial cellular entry mediator or as a bridging protein for rickettsial accumulation with an additional host cell surface protein. As previously noted, the C-terminal lysyl residues of histone H2B serve as a plasminogen recognition binding site (Das *et al.*, 2007; Herren *et al.*, 2006; Miles *et al.*, 1991). In addition, the host plasminogen was reported as the dissemination/invasion activator for other tick-borne pathogens, i.e. *Borrelia burgdorferi* (Coleman *et al.*, 1997) and *Borrelia hermsii* (Rossmann *et al.*, 2007). Although the histone–plasminogen interaction occurs in a vertebrate infection system, further research should determine if additional arthropod-derived factors are involved.

Consistent with the current study, arthropod-derived histone has also been associated with other obligate intracellular bacteria closely related to *Rickettsia*. *Wolbachia* aggregates with *Drosophila* histone protein during sperm–egg cytoplasmic incompatibility, facilitating maternal transmission (Poinsot *et al.*, 2003). It is not clear if rickettsiae manipulate the reproductive function in blood-feeding arthropods; however, it is reasonable to suggest that two closely related organisms such as *Wolbachia* and *Rickettsia*, employing the same survival strategy (i.e. vertical transmission by arthropods), would utilize a conserved molecule such as histone. The ability of organisms such as *Rickettsia* to persist in nature via the perpetual infection of a variety of blood-feeding arthropods is testimony to a successful approach to survival in the absence of a complex amplification-dependent transmission cycle. As the specific mechanisms of infection become clearer, the selection of a ubiquitous and abundant molecule such as histone would be a viable approach for *Rickettsia* to continue to spread itself to a variety of host cell types. Continued studies on the infection mechanisms of arthropod hosts by *Rickettsia* will facilitate a better understanding of the ecology and epidemiology of vector-borne rickettsial diseases.

## Abbreviations

- Co-IP, co-immunoprecipitation
- CpB, carboxypeptidase B
- PPIase, peptidyl-prolyl *cis-trans* isomerise
- qPCR, quantitative real-time PCR
- RNAi, RNA-mediated interference

## References

[r1] Amsel, A. D., Rathaus, M., Kronman, N. & Cohen, H. Y. (2008). Regulation of the proapoptotic factor Bax by Ku70-dependent deubiquitylation. Proc Natl Acad Sci U S A 105, 5117–5122. 18362350 10.1073/pnas.0706700105 PMC2278173

[r2] Anacker, R. L., Mann, R. E. & Gonzales, C. (1987). Reactivity of monoclonal antibodies to *Rickettsia rickettsii* with spotted fever and typhus group rickettsiae. J Clin Microbiol 25, 167–171. 2432081 10.1128/jcm.25.1.167-171.1987 PMC265851

[r3] Baake, M., Doenecke, D. & Albig, W. (2001). Characterisation of nuclear localisation signals of the four human core histones. J Cell Biochem 81, 333–346. 11241673

[r4] Behrens, S., Maier, R., de Cock, H., Schmid, F. X. & Gross, C. A. (2001). The SurA periplasmic PPIase lacking its parvulin domains functions in vivo and has chaperone activity. EMBO J 20, 285–294. 11226178 10.1093/emboj/20.1.285 PMC140197

[r5] Bolton, S. J. & Perry, V. H. (1997). Histone H1; a neuronal protein that binds bacterial lipopolysaccharide. J Neurocytol 26, 823–831. 9482158 10.1023/a:1018574600961

[r6] Borun, T. W., Scharff, M. D. & Robbins, E. (1967). Rapidly labeled, polyribosome-associated RNA having the properties of histone messenger. Proc Natl Acad Sci U S A 58, 1977–1983. 5237492 10.1073/pnas.58.5.1977 PMC223893

[r7] Coleman, J. L., Gebbia, J. A., Piesman, J., Degen, J. L., Bugge, T. H. & Benach, J. L. (1997). Plasminogen is required for efficient dissemination of *B. burgdorferi* in ticks and for enhancement of spirochetemia in mice. Cell 89, 1111–1119. 9215633 10.1016/s0092-8674(00)80298-6

[r8] Das, R., Burke, T. & Plow, E. F. (2007). Histone H2B as a functionally important plasminogen receptor on macrophages. Blood 110, 3763–3772. 17690254 10.1182/blood-2007-03-079392 PMC2077321

[r9] de la Fuente, J., Blouin, E. F., Manzano-Roman, R., Naranjo, V., Almazan, C., Perez de la Lastra, J. M., Zivkovic, Z., Jongejan, F. & Kocan, K. M. (2007). Functional genomic studies of tick cells in response to infection with the cattle pathogen, *Anaplasma marginale*. Genomics 90, 712–722. 17964755 10.1016/j.ygeno.2007.08.009

[r10] Emelyanov, V. V. & Demyanova, N. G. (1999). Nucleotide sequence of the gene and features of the major outer membrane protein of a virulent *Rickettsia prowazekii* strain. Biochemistry (Mosc) 64, 494–503. 10381609

[r11] Featherstone, C. & Jackson, S. P. (1999). Ku, a DNA repair protein with multiple cellular functions? Mutat Res 434, 3–15. 10377944 10.1016/s0921-8777(99)00006-3

[r12] Gasteiger, E., Hoogland, C., Gattiker, A., Duvaud, S., Wilkins, M. R., Appel, R. D. & Bairoch, A. (2005). Protein identification and analysis tools on the ExPASY serverThe Proteomics Protocols Handbook, pp. 571–607. Edited by Walker, J. M.. Totowa, NJ. : Humana Press.

[r13] Hackstadt, T. (1996). The biology of rickettsiae. Infect Agents Dis 5, 127–143. 8805076

[r14] Heinzen, R. A., Hayes, S. F., Peacock, M. G. & Hackstadt, T. (1993). Directional actin polymerization associated with spotted fever group rickettsia infection of Vero cells. Infect Immun 61, 1926–1935. 8478082 10.1128/iai.61.5.1926-1935.1993 PMC280785

[r15] Heinzen, R. A., Grieshaber, S. S., Van Kirk, L. S. & Devin, C. J. (1999). Dynamics of actin-based movement by *Rickettsia rickettsii* in Vero cells. Infect Immun 67, 4201–4207. 10417192 10.1128/iai.67.8.4201-4207.1999 PMC96725

[r16] Herren, T., Burke, T. A., Das, R. & Plow, E. F. (2006). Identification of histone H2B as a regulated plasminogen receptor. Biochemistry 45, 9463–9474. 16878981 10.1021/bi060756w

[r17] Holers, V. M. & Kotzin, B. L. (1985). Human peripheral blood monocytes display surface antigens recognized by monoclonal antinuclear antibodies. J Clin Invest 76, 991–998. 3876357 10.1172/JCI112100 PMC423964

[r18] Kedes, L. H. (1979). Histone genes and histone messengers. Annu Rev Biochem 48, 837–870. 112914 10.1146/annurev.bi.48.070179.004201

[r19] Khan, I. U., Wallin, R., Gupta, R. S. & Kammer, G. M. (1998). Protein kinase A-catalyzed phosphorylation of heat shock protein 60 chaperone regulates its attachment to histone 2B in the T lymphocyte plasma membrane. Proc Natl Acad Sci U S A 95, 10425–10430. 9724719 10.1073/pnas.95.18.10425 PMC27910

[r20] Kurtti, T. J., Mattila, J. T., Herron, M. J., Felsheim, R. F., Baldridge, G. D., Burkhardt, N. Y., Blazar, B. R., Hackett, P. B., Meyer, J. M. & Munderloh, U. G. (2008). Transgene expression and silencing in a tick cell line: a model system for functional tick genomics. Insect Biochem Mol Biol 38, 963–968. 18722527 10.1016/j.ibmb.2008.07.008 PMC2581827

[r21] Li, H. & Walker, D. H. (1998). rOmpA is a critical protein for the adhesion of *Rickettsia rickettsii* to host cells. Microb Pathog 24, 289–298. 9600861 10.1006/mpat.1997.0197

[r22] Lundemose, A. G., Kay, J. E. & Pearce, J. H. (1993). *Chlamydia trachomatis* Mip-like protein has peptidyl-prolyl *cis/trans* isomerase activity that is inhibited by FK506 and rapamycin and is implicated in initiation of chlamydial infection. Mol Microbiol 7, 777–783. 7682281 10.1111/j.1365-2958.1993.tb01168.x

[r23] Macaluso, K. R., Mulenga, A., Simser, J. A. & Azad, A. F. (2003). Differential expression of genes in uninfected and *Rickettsia*-infected *Dermacentor variabilis* ticks as assessed by differential-display PCR. Infect Immun 71, 6165–6170. 14573632 10.1128/IAI.71.11.6165-6170.2003 PMC219596

[r24] Macaluso, K. R., Mulenga, A., Simser, J. A. & Azad, A. F. (2006). Characterization of *Dermacentor variabilis* molecules associated with rickettsial infection. Ann N Y Acad Sci 1078, 384–388. 17114746 10.1196/annals.1374.076

[r25] Martinez, J. J. & Cossart, P. (2004). Early signaling events involved in the entry of *Rickettsia conorii* into mammalian cells. J Cell Sci 117, 5097–5106. 15383620 10.1242/jcs.01382

[r26] Martinez, J. J., Seveau, S., Veiga, E., Matsuyama, S. & Cossart, P. (2005). Ku70, a component of DNA-dependent protein kinase, is a mammalian receptor for *Rickettsia conorii*. Cell 123, 1013–1023. 16360032 10.1016/j.cell.2005.08.046

[r27] Marzluff, W. F. & Duronio, R. J. (2002). Histone mRNA expression: multiple levels of cell cycle regulation and important developmental consequences. Curr Opin Cell Biol 14, 692–699. 12473341 10.1016/s0955-0674(02)00387-3

[r28] Miles, L. A., Dahlberg, C. M., Plescia, J., Felez, J., Kato, K. & Plow, E. F. (1991). Role of cell-surface lysines in plasminogen binding to cells: identification of alpha-enolase as a candidate plasminogen receptor. Biochemistry 30, 1682–1691. 1847072 10.1021/bi00220a034

[r29] Mulenga, A., Macaluso, K. R., Simser, J. A. & Azad, A. F. (2003). Dynamics of *Rickettsia*–tick interactions: identification and characterization of differentially expressed mRNAs in uninfected and infected *Dermacentor variabilis*. Insect Mol Biol 12, 185–193. 12653940 10.1046/j.1365-2583.2003.00400.x

[r30] Munderloh, U. G. & Kurtti, T. J. (1989). Formulation of medium for tick cell culture. Exp Appl Acarol 7, 219–229. 2766897 10.1007/BF01194061

[r31] Munderloh, U. G. & Kurtti, T. J. (1995). Cellular and molecular interrelationships between ticks and prokaryotic tick-borne pathogens. Annu Rev Entomol 40, 221–243. 7810987 10.1146/annurev.en.40.010195.001253

[r32] Poinsot, D., Charlat, S. & Mercot, H. (2003). On the mechanism of Wolbachia-induced cytoplasmic incompatibility: confronting the models with the facts. Bioessays 25, 259–265. 12596230 10.1002/bies.10234

[r33] Pornwiroon, W., Pourciau, S. S., Foil, L. D. & Macaluso, K. R. (2006). *Rickettsia felis* from cat fleas: isolation and culture in a tick-derived cell line. Appl Environ Microbiol 72, 5589–5595. 16885313 10.1128/AEM.00532-06 PMC1538700

[r34] Pornwiroon, W., Bourchookarn, A., Paddock, C. D. & Macaluso, K. R. (2009). Proteomic analysis of *Rickettsia parkeri* strain portsmouth. Infect Immun 77, 5262–5271. 19797064 10.1128/IAI.00911-09 PMC2786434

[r35] Prabhakar, B. S., Allaway, G. P., Srinivasappa, J. & Notkins, A. L. (1990). Cell surface expression of the 70-kD component of Ku, a DNA-binding nuclear autoantigen. J Clin Invest 86, 1301–1305. 2212014 10.1172/JCI114838 PMC296862

[r36] Qiu, H. & Wang, Y. (2008). Quantitative analysis of surface plasma membrane proteins of primary and metastatic melanoma cells. J Proteome Res 7, 1904–1915. 18410138 10.1021/pr700651b PMC4704867

[r37] Ramakrishnan, V. G., Aljamali, M. N., Sauer, J. R. & Essenberg, R. C. (2005). Application of RNA interference in tick salivary gland research. J Biomol Tech 16, 297–305. 16522848 PMC2291763

[r38] Reif, K. E. & Macaluso, K. R. (2009). Ecology of *Rickettsia felis*: a review. J Med Entomol 46, 723–736. 19645274 10.1603/033.046.0402 PMC12794530

[r39] Rossmann, E., Kraiczy, P., Herzberger, P., Skerka, C., Kirschfink, M., Simon, M. M., Zipfel, P. F. & Wallich, R. (2007). Dual binding specificity of a *Borrelia hermsii*-associated complement regulator-acquiring surface protein for factor H and plasminogen discloses a putative virulence factor of relapsing fever spirochetes. J Immunol 178, 7292–7301. 17513779 10.4049/jimmunol.178.11.7292

[r40] Silverman, D. J. & Wisseman, C. L., Jr (1979). In vitro studies of rickettsia–host cell interactions: ultrastructural changes induced by *Rickettsia rickettsii* infection of chicken embryo fibroblasts. Infect Immun 26, 714–727. 121115 10.1128/iai.26.2.714-727.1979 PMC414674

[r41] Simser, J. A., Mulenga, A., Macaluso, K. R. & Azad, A. F. (2004). An immune responsive factor D-like serine proteinase homologue identified from the American dog tick, *Dermacentor variabilis*. Insect Mol Biol 13, 25–35. 14728664 10.1111/j.1365-2583.2004.00455.x

[r42] Sunyakumthorn, P., Bourchookarn, A., Pornwiroon, W., David, C., Barker, S. A. & Macaluso, K. R. (2008). Characterization and growth of polymorphic *Rickettsia felis* in a tick cell line. Appl Environ Microbiol 74, 3151–3158. 18359823 10.1128/AEM.00025-08 PMC2394910

[r43] Tai, Y. T., Podar, K., Kraeft, S. K., Wang, F., Young, G., Lin, B., Gupta, D., Chen, L. B. & Anderson, K. C. (2002). Translocation of Ku86/Ku70 to the multiple myeloma cell membrane: functional implications. Exp Hematol 30, 212–220. 11882358 10.1016/s0301-472x(01)00786-x

[r44] Uchiyama, T., Kawano, H. & Kusuhara, Y. (2006). The major outer membrane protein rOmpB of spotted fever group rickettsiae functions in the rickettsial adherence to and invasion of Vero cells. Microbes Infect 8, 801–809. 16500128 10.1016/j.micinf.2005.10.003

[r45] Watson, K., Gooderham, N. J., Davies, D. S. & Edwards, R. J. (1999). Nucleosomes bind to cell surface proteoglycans. J Biol Chem 274, 21707–21713. 10419482 10.1074/jbc.274.31.21707

[r46] Weiss, E. (1982). The biology of rickettsiae. Annu Rev Microbiol 36, 345–370. 6756292 10.1146/annurev.mi.36.100182.002021

[r47] Whitworth, T., Popov, V. L., Yu, X. J., Walker, D. H. & Bouyer, D. H. (2005). Expression of the *Rickettsia prowazekii* *pld* or *tlyC* gene in *Salmonella enterica* serovar Typhimurium mediates phagosomal escape. Infect Immun 73, 6668–6673. 16177343 10.1128/IAI.73.10.6668-6673.2005 PMC1230948

[r48] Zanettii, A. S., Pornwiroon, W., Kearney, M. T. & Macaluso, K. R. (2008). Characterization of rickettsial infection in *Amblyomma americanum* (Acari: Ixodidae) by quantitative real-time polymerase chain reaction. J Med Entomol 45, 267–275. 18402143 10.1603/0022-2585(2008)45[267:coriia]2.0.co;2

[r49] Zavala-Castro, J. E., Small, M., Keng, C., Bouyer, D. H., Zavala-Velazquez, J. & Walker, D. H. (2005). Transcription of the *Rickettsia felis* *ompA* gene in naturally infected fleas. Am J Trop Med Hyg 73, 662–666. 16222005 PMC1440719

[r50] Zhu, G., Chen, H., Choi, B. K., Del, P. F. & Schifferli, D. M. (2005). Histone H1 proteins act as receptors for the 987P fimbriae of enterotoxigenic *Escherichia coli*. J Biol Chem 280, 23057–23065. 15840569 10.1074/jbc.M503676200

[r51] Zlatanova, J. S., Srebreva, L. N., Banchev, T. B., Tasheva, B. T. & Tsanev, R. G. (1990). Cytoplasmic pool of histone H1 in mammalian cells. J Cell Sci 96, 461–468. 2229196 10.1242/jcs.96.3.461

